# Editorial: Regulation of lipid metabolism in adipose tissue and skeletal muscle

**DOI:** 10.3389/fphys.2022.1010578

**Published:** 2022-11-04

**Authors:** Zhihao Jia, Tizhong Shan

**Affiliations:** ^1^ Cambridge-Suda Genomic Resource Center, Suzhou Medical College, Soochow University, Suzhou, China; ^2^ Department of Animal Sciences, Purdue University, West Lafayette, IN, United States; ^3^ College of Animal Sciences, Hangzhou, China; ^4^ Key Laboratory of Molecular Animal Nutrition, Ministry of Education, Zhejiang University, Hangzhou, China; ^5^ Key Laboratory of Animal Feed and Nutrition of Zhejiang Province, Hangzhou, China

**Keywords:** lipid metabolism, adipose tissue, skeletal muscle, metabolic disorder, T2D

## Introduction

Obesity due to excessive deposition of lipids in adipose tissue (AT) has become a global epidemic. Obesity and its increased risks of chronic diseases including Type 2 Diabetes (T2D), chronic inflammation, hypertension, and cancer have posed formidable challenges to human health. Adipocytes are highly plastic and can uptake, esterificate, and store excess lipids in the form of triacylglycerols (TAG) within lipid droplets (LDs), as well as undergo lipolysis to provide energy when nutrition is limited. Non-AT such as skeletal muscle, which has a comparatively high capacity for fatty acid oxidation to generate energy, becomes dysfunctional with “lipid overload”. Thus, the dysregulation of lipid metabolism in skeletal muscle highly contributes to obese-related insulin resistance. Thus, understanding how lipid storage and utilization are regulated in AT and skeletal muscle is critical for the development of therapeutics to overcome obesity. Indeed, interventions that increase AT/muscle fatty acid oxidation and/or limit lipid storage have been postulated as therapies for treating obesity-related conditions. Besides, lipid metabolism in AT and skeletal muscle are also closely associated with growth performance, meat quality, and reproduction in livestock farming. Therefore, this Research Topic aims to compile articles that expand our understanding of the lipid metabolism processes within the AT and skeletal muscle.

## Beige/brown adipocyte thermogenesis

There are three types of adipocytes: brown adipocyte, white adipocyte, and beige adipocyte. Brown and beige adipocyte dissipate glucose and fatty acid (FA) to generate heat and play important roles in cold- and diet-induced thermogenesis (Kajimura et al., 2015). Therefore, genetically and pharmaceutically activation of beige/brown adipocyte has drawn great attention in the treatment of metabolic diseases. Li et al. analyze and compare the transcriptomics of AT after chronic cold exposure and β3-AR agonist CL316,243 (CL) administration. The results reveal that cold and CL treatment both upregulates genes in lipid metabolism and TCA cycle pathways. CL treatment uniquely activates genes in carbohydrate mobilization, while cold uniquely promotes genes in glycolipid and specific amino acids metabolism. In addition, cold and CL treatment could separately suppress different inflammatory pathways. In particular, cold specifically inhibits fibrotic programs.

Lipoprotein lipase (LPL) expressed by brown adipocyte play a central role in lipid metabolism by processing triglyceride-rich lipoproteins (TRL). Thiemann et al. observe that *Lpl* is also expressed by endothelial cells in cold-activated brown adipose tissue (BAT). By using a VE-cadherin (*Cdh5*)-Cre to drive the knockout of *lpl*, they demonstrate that endothelial *Lpl* is dispensable for lipoprotein handling and adaptive thermogenesis. The authors hypothesize that a compensatory high expression of LPL in brown adipocyte may enhance BAT TRL disposal in this model, but the mechanism remains unclear.

In another interesting review, Bruder et al. discuss the potential physiological purposes of postnatal AT remodeling. They state that postnatal AT remodeling is an ideal model process to investigate beige/brown adipocyte thermogenesis since there is less external manipulation.

## Epigenetic regulation

In the condition of overnutrition, AT is able to expand in two ways: Hyperplasia that is dependent on increasing adipocyte number through adipogenesis and hypertrophy by enlargement of adipocyte cell size (Ghaben and Scherer, 2019). Notably, targeting adipogenesis is now emerging for the treatment of obesity. Interest in the epigenetic roles of non-coding RNAs during adipogenesis has been growing rapidly (Sun et al., 2013). Zhang et al. review the recent advances in long non-coding RNAs (lncRNAs) that regulate AT development and metabolism. In particular, they summarize the potential involvement of lncRNAs in metabolic disorders and prospect the potential of targeting lncRNAs in the treatment of obesity and metabolic diseases by using antisense oligonucleotides (ASO). Besides non-coding RNA, RNA m6A methylation (m6A) also plays many aspects in various diseases (He and He, 2021). Wang et al. introduce the up-to-date research advances between m6A and lipid metabolism, especially those within non-alcoholic fatty liver disease (NAFLD), diabetes, and cardiovascular diseases. This review provides insights into the possibilities that modify RNA m6A methylation in the fight against metabolic diseases.

Circular RNA (circRNA) is a newly discovered type of non-coding RNAs that participates in numerous cellular processes. Zhang et al. perform RNA-sequencing and identify 3,711 circRNAs at different stages of adipogenesis (preadipocyte, differentiating preadipocyte, and mature adipocyte). They find that many circRNAs share similar expression patterns with their parental genes. In addition, they discover multiple microRNA (miRNA) binding sites from the differential expressed circRNAs, which indicates that crircRNAs may act as miRNA sponges. However, the function of circRNA during adipogenesis requires to be further evaluated.

## Disease-relevant study

Besides its role in energy storage, AT also contributes to systemic metabolism as an endocrine organ, especially through the secretion of adipokines (Fasshauer and Blüher, 2015). Ji et al. review the role of retinol-binding protein 4 (RBP4) in lipid metabolism and highlight the emerging importance of RBP4 with cardiovascular diseases (CVDs). By comparing published datasets, they predict that RBP4 might not only be a new biomarker for CVD, but targeting RBP4 preserves therapeutic potentials.

For years, scientists have been investigating antihyperglycemic drugs to protect against tissue damage in T2D (Verma and McMurray, 2018). The administration of a sodium-glucose cotransporter 2 inhibitor (SGLT2i) has been proven as a candidate therapy for hyperglycemia in T2D patients (Chao and Henry, 2010). Nagayama et al. extend the application of SGLT2i to incomplete acquired lipodystrophy. They report a case wherein a patient is treated with a combination of metreleptin supplementation and SGLT2i to improve hyperglycemia and insulin sensitivity. This study provides the potential benefits of using SGLT2i for non-obese diabetic patients.

## Skeletal muscle metabolism

The skeletal muscle is the largest metabolic organ in the body and is extremely important for energy homeostasis (Sheffield-Moore and Urban, 2004). Antony et al. investigate the role of UCHL1 in skeletal muscle lipid metabolism. They observe that in fasting or glucose starvation conditions, UCHL1 protein levels are decreased in both skeletal muscle and differentiated myotubes. Muscle-specific *Uchl1* knockout reduces lipid content and improves glucose tolerance, which may be correlated with the stabilization of Perilipin 2. In another research article on patients with spinal cord injury (SCI), Goldsmith et al. show that increased visceral adiposity, and inflammatory signaling, as well as reduced testosterone levels, predict mitochondrial dysfunction in these patients. However, due to the restriction in patient size of the current study, it remains unclear the complex causal relationships among those factors with chronic SCI. To better address the limitations more effectively, a large multi-centered trial is highly warranted.

## Comparative studies

In addition to its association with human health, lipid metabolism in AT and skeletal muscle are also extremely popular among non-medical researchers, especially for those working in agriculture. Conserved signaling pathways that govern adipose and skeletal muscle development and metabolism have been investigated in agricultural animals, such as liver kinase B1 (LKB1) (Shan et al., 2014; Shan et al., 2016). Xiong et al. report a role of LKB1 in goat intramuscular preadipocytes differentiation *via* the focal adhesion kinase (FAK) pathway. The results indicate that targeting LKB1 might be a potential strategy to manipulate intramuscular fat deposition to improve the meat quality of goat. Pig farming requires stale temperature control as piglets lack the ability for thermogenesis. For this purpose, Xu et al. explore the effect of acute cold exposure on carcass indicators, enzyme activity, fatty acid composition, and gene expression profiles in the longissimus dorsi muscle (LDM) of growing-finishing pigs. The results suggest that chronic cold exposure significantly changes fatty acid profile and lipid metabolism, which emphasizes the importance of pre-slaughter temperature conditions in the fatty acid metabolism of pork. However, no difference is found in the content of amino acids, which needs to be further investigated in a longer time of cold treatment.

## Conclusion

In sum, the 12 articles in this Research Topic are representative of the depth content of lipid metabolism in AT and skeletal muscle, as well as their application in metabolic diseases. Results from the aforementioned articles could help better unravel the selective and specific biomarkers and therapeutic targets of various metabolic diseases.
